# Supplementary material for: 8-Week Supplementation of 2S-Hesperidin Modulates Antioxidant and Inflammatory Status after Exercise until Exhaustion in Amateur Cyclists
Source: Antioxidants (Basel). 2021 Mar 11;10(3):432. doi: 10.3390/antiox10030432 (PMC8000657; doi:10.3390/antiox10030432)
Supplement: Supplementary file 1 [file antioxidants-10-00432-s001.pdf]

Supplementary file 1. Methodology oxidative, antioxidant and inflammatory markers.

TBARS (Lipoperoxidation biomarker)

Thiobarbituric acid reactive substances (TBARS) are by-products of the oxidative degradation of lipids by reactive oxygen species (lipid peroxidation), which is commonly used as an oxidative stress marker. The methodology consists of isolating the plasma lipid fraction by lipid precipitation with phosphotungstic acid, followed by a reaction with thiobarbituric acid (TBA) that forms an adduct, allowing for detection using UV-VIS spectrophotometer at a wavelength of 532 nm. The assay involves the reaction of malondialdehyde (MDA), a product of lipid peroxidation, with thiobarbituric acid (TBA) under high temperature and acidic conditions to form an MDA-TBA complex that can be measured colourimetrically. The coefficient of variation between replicas had to be less than or equal to 4.6 %.

Catalase (CAT)

CAT activity was determined using a UV-VIS spectrophotometer. The absorbance of H2O2 decreases at 240 nm proportional to its decomposition, so that the concentration of H2O2 is critical in this determination. The decrease in absorbance per unit time is the measure of catalase activity. This is expressed in per sec per gram of haemoglobin. The coefficient of variation between replicas must be less than or equal to 4.9 %.

Superoxide dismutase (SOD)

SOD activity was measured using an SD125 Ransod kit (Randox Ltd. Crumlin, United Kingdom). This method consists of the use of xanthine and xanthine oxidase to produce superoxide anion (O2·-), which responds with the 2-(4-iodophenyl)-3-(4-nitrophenol)-5-phenyltetrazolium chloride (INT) reactive and forms a red complex detectable at 420 nm. The SOD activity is then quantified by measuring the degree of inhibition of this reaction. The coefficient of variation between replicas must be less than or equal to 5.1 %.

Glutathione reduced (GSH)

The GSH was determined from whole blood, which was treated with perchloric acid to a final concentration of 6%, obtaining the supernatant after vortexing and centrifuging at 10,000 rpm for 10 minutes. After collecting the supernatants in vials, it was quantified using high-performance liquid chromatography (HPLC) with a

Waters ODS S5 NH2 Column (0.052, 25 cm) for separation purposes. The coefficient of variation for GSH between replicas must be less than or equal to 4.1 %.

Hemoxigenasa 1 (HO-1)

HO-1 ELISA kit applies the competitive enzyme immunoassay technique by utilizing an anti-HO-1 antibody and an HO-1-HRP conjugate. The assay sample and buffer are incubated together with HO-1-HPR conjugate in a pre-coated plate for one hour. After the incubation period, the wells are decanted and washed five times. The wells are then incubated with a substrate for HRP enzyme. The product of the enzyme-substrate reaction forms a blue colored complex. Finally, a stop solution yellow is formed. The intensity of the color is measured spectrophotometrically at 450 nm in a microplate reader. The intensity of the color is inversely proportional to the HO-1 concentration, since HO-1 from samples and HO-1-HRP conjugate compete for the anti-HO-1 antibody binding site. As there are a set number of sites and as more sites are occupied by HO-1 from the sample, there are fewer sites left to bind to the HO-1-HRP conjugate. A standard curve is plotted relating the intensity of the color (O.D.) to the concentration of standards. The HO-1 concentration in each sample is interpolated from this standard curve. The coefficient of variation between replicas must be less than or equal to 4.9 %.

Interleukin 6 (IL-6)

The DRG IL-6-ELISA is a solid phase Enzyme Amplified Sensitivity Immunoassay performed on microtiterplate. The assay uses monoclonal antibodies (MAbs) directed against distinct epitopes of IL-6. Calibrators and samples react with the capture monoclonal antibody (MAb 1) coated on a microtiter well and with a monoclonal antibody (MAb 2) labelled with horseradish peroxidase (HRP). After an incubation period that allows for the formation of a sandwich (coated MAb 1 –human IL-6 – MAb 2 – HRP), the microtiterplate is washed to remove unbound enzyme labelled antibody. Bound enzyme-labelled antibody is measured through a chromogenic reaction. Chromogenic solution (TMB) is added and incubated. The reaction is stopped with the addition of a Stop Solution, and the microtiterplate is then read at the appropriate wavelength. The amount of substrate turnover is determined colourimetrically by measuring the absorbance, which is proportional to the IL-6 concentration. A calibration curve is plotted and IL-6 concentration in samples is determined by interpolation from the calibration curve. The use of the EASIA reader (linearity up to 3 OD units) and a sophisticated data reduction method (polychromatic data reduction) result in a high sensitivity in the low range and in an extended calibration range. The coefficient of variation between replicas must be less than or equal to 4.4 %.

Tumoral necrosis factor α (TNFα)

The DRG TNF-α-ELISA is a solid phase Enzyme Amplified Sensitivity Immunoassay performed on microtiterplate. The assay uses monoclonal antibodies (MAbs) directed against distinct epitopes of TNF-α. Standards and samples react with the capture monoclonal antibody (MAb 1), coated on microtiter well and with a monoclonal antibody (MAb 2) labelled with horseradish peroxidase (HRP). After an incubation period that allows for the formation of a sandwich (coated MAb 1 – human TNF-α – MAb 2 – HRP), the microtiterplate is washed to remove unbound enzyme labelled antibody. Bound enzyme-labelled antibody is measured through a chromogenic reaction. Chromogenic solution (TMB) is added and incubated. The reaction is stopped with the addition of a Stop Solution, and the microtiterplate is then read at the appropriate wavelength. The amount of substrate turnover is determined colourimetrically by measuring the absorbance, which is proportional to the TNF-α concentration.

A calibration curve is plotted, and TNF-α concentration in samples is determined by interpolation from the calibration curve. The use of the EASIA reader (linearity up to 3 OD units) and a sophisticated data reduction method (polychromatic data reduction) result in a high sensitivity in the low range and in an extended calibration range. For guidance, the results of 30 serum samples from apparently healthy persons with low CRP levels, ranged between 4.6 and 12.4 pg/ml, were used. The coefficient of variation between replicas must be less than or equal to 6.4 %.

Monocyte chemoattractant protein 1 (MCP-1)

An anti-human MCP-1 coating antibody is absorbed onto microwells. Human MCP-1 present in a sample or standard binds to antibodies absorbed to the microwells, and the horseradish peroxidase (HRP)-conjugated anti-human MCP-1 antibody is added and binds to human MCP-1 captured by the first antibody. Following incubation, unbound HRP-conjugated anti-human is removed during a wash step, and substrate solution reactive with HRP is added to the wells.

A coloured product is formed in proportion to the amount of human MCP-1 present in the sample or standard. The reaction is terminated by addition of acid and absorbance is measured at 450 nm. A standard curve is prepared from 7 human MCP-1 standard dilutions and human MCP-1 concentration determined. The limit of detection of human MCP-1, defined as the analyte concentration resulting in an absorbance significantly higher than that of the dilution medium (mean plus 2 standard deviations), was determined to be 2.3 pg/mL (mean of 6 independent assays). The coefficient of variation between replicas must be less than or equal to 4.7 %.
